# Image-guided biopsy of breast lesions—when to use what biopsy technique: the United States and Canadian perspective

**DOI:** 10.1186/s13244-025-02155-7

**Published:** 2025-12-22

**Authors:** Katja Pinker, Christopher Comstock, Jessica W. T. Leung, Roberto LoGullo, Habib Rahbar, Jean M. Seely, Isabelle Trop, Janice Sung

**Affiliations:** 1 Division of Breast Imaging, Department of Radiology, Columbia University Irving Medical Center, New York, NY USA; 2 Department of Radiology, Weill Cornell Medicine/New York-Presbyterian Hospital, New York, NY USA; 3 Department of Breast Imaging, Division of Diagnostic Imaging, The University of Texas MD Anderson Cancer Center, Houston, TX USA; 4 Department of Radiology, University of Washington, Fred Hutchinson Cancer Center, Seattle, WA USA; 5 Department of Radiology, The Ottawa Hospital, General Campus, Ottawa, ON Canada; 6 Department of Radiology, Centre Hospitalier de Montréal, CHUM, Montréal, QC Canada

The study this opinion refers to, “Image-guided biopsy of breast lesions—when to use what biopsy technique,” presents a statement by an international panel of experts in breast imaging and interventions to address the scarcity of evidence-based literature on optimal biopsy approaches in breast radiology [1].

While the USA and Canada were not part of the voting panel, an expert radiologist group from the United States and Canada was invited to review these statements for comments and endorsement. This expert radiologist group endorses the manuscript with specific considerations.

The experts agree that in breast lesions (non-axillary lymph nodes) visible on sonography, ultrasound-guided biopsy is the preferred technique, and core needle biopsy (CNB) is preferred over fine needle aspiration (FNA). FNA of breast lesions is reserved for aspiration of simple and complicated cysts, fluid collections such as abscesses, post-operative seromas, or peri-implant fluid, and lesions that are not amenable to CNB due to their location. FNA may be a primary option at centers where cytopathology is of exceptional quality and there is commitment to on-site cytopathology support to allow for immediate conversion to CNB when atypia is present. However, this level of support and expertise is uncommon, and in general, CNB is highly preferred due to its rare yield of “non-diagnostic” samples and ability to facilitate important immunohistochemistry on samples that reveal malignancy.

In the setting of recurring abscesses or mastitis not responding to antibiotic treatment, CNB can be considered to rule out inflammatory breast cancer as well as to rule in other types of inflammatory mastitis, such as granulomatous mastitis.

CNB is preferred over vacuum-assisted biopsy (VAB) for sonographic masses of any size that are well visualized on ultrasound (i.e., USA and Canadian practice standards do not use a size cutoff, such as over 5 mm, that is recommended in the international statement). Rather, the selection of biopsy techniques for US-guided procedures is driven by lesion visibility, lesion type, and sampling indication. VAB is preferred for sonographic masses that are not well-defined (i.e., non-mass lesions at ultrasound) and without a correlate on other imaging modalities, as well as for complex cystic and solid lesions with small (< 5 mm) solid parts where there is concern that CNB might not allow for adequate sampling of the solid portion of the lesion (e.g., very small solid component or concern that solid portion will not be visible upon rupture of the cystic component during biopsy).

In contrast to tissue sampling of the primary breast lesion, where CNB is recommended over FNA, axillary lymph nodes may be biopsied using either FNA or CNB based on institutional or physician preference. This recommendation for biopsy method selection based on institutional or physician preference is reflective of the fact that, in the international statement, consensus was not reached, with 50% of the panel preferring FNA, and 50% preferring CNB. As the international panel mentions, when the question is to determine whether a node is metastatic or not from a known breast cancer, FNA may be appropriate, whereas larger samples with CNB will be most useful to diagnose alternate processes involving an abnormal node. It should be noted that in cases where an axillary lesion is likely a very abnormal axillary lymph node but that the imaging are so abnormal morphologically (e.g., completely rounded and no longer demonstrating a reniform shape) such that it is plausible that the lesion could represent a separate process, CNB may be particularly helpful to help confirm the presence of some residual lymph node architecture.

The experts agree that in lesions only visible on mammography, DBT or MRI, the preferred method of choice for biopsy is VAB. CNB is only reserved for cases when VAB is not available. For MRI lesions, there is a recommendation for image-guided localization and excisional breast biopsy only if percutaneous biopsy is not feasible. For architectural distortions and calcifications, the preferred biopsy method of choice is mammography-guided VAB if the lesion is not visible with ultrasound. If a sonographic correlate is visible, US-guided VAB biopsy should be performed. In biopsies of microcalcifications, a specimen radiograph should be obtained regardless of the biopsy technique used to confirm adequate sampling.

Recommendations for high-risk lesions detected on either CNB or VAB are outlined in Table 1. It has to be noted that these can slightly vary between institutions and may also be risk-based. In this respect, the experts disagree with the recommendation for VAB extensions, i.e., extended vacuum-assisted biopsy (EVAB) for unambiguous lesion classification and vacuum-assisted excision (VAE) for complete lesion removal in high-risk lesions. Re-biopsy should be considered for inconclusive findings or suspected under-sampling, with a preference for VAB over CNB in this scenario.

**Table 1:** Recommendations for high-risk lesions detected on either CNB or VAB

Core needle biopsy lesion	Excision recommended
Intraductal papilloma	
No atypia	No
No atypia, symptomatic	Yes
Atypia	Yes
Papillary lesion	
Atypia	Yes
Radial scar/complex sclerosing lesion	
No atypia	No
Atypia	Yes
Mucocele-like lesion/stromal mucin	
No atypia	No
Atypia	Yes
Lobular neoplasia	
Classic LCIS and ALH	No
Non-classic LCIS (pleomorphic/pleomorphic features and Florid)	Yes
Flat epithelial atypia (FEA)	
Personal history of breast cancer	Depending on institutional practice
No personal history of breast cancer	No
Atypical ductal hyperplasia (ADH)	
Focal (1 focus, < 2 mm)	Yes^a^
Non-focal	Yes

To avoid excision, rad-path concordance must be present

“Atypia” refers to ductal atypia (ADH and/or DCIS)^b^

*CNB* core needle biopsy*, VAB* vacuum-assisted biopsy

^a^ If clinical need arises, imaging follow-up can be considered

^b^ Lesions associated with ALH or classic LCIS do NOT require excision

No re-biopsy or excision is recommended for classic lobular neoplasia and FEA in patients without a personal history of breast cancer on either CNB or VAB. For phyllodes tumors (benign or borderline) or fibroepithelial lesions, particularly if mentioned with increased stromal cellularity found with initial CNB or VAB, surgical excision is recommended.

For DCIS diagnosed with CNB or VAB, surgical excision is recommended. Additional imaging with MRI or contrast-enhanced mammography in the setting of high-grade cancers, invasive lobular carcinoma, women with very dense breasts, those with strong family history, and young patients should be considered for assessment of the extent of disease.

At present, minimally invasive image-guided interventions for the treatment of breast cancers are reserved for women who are unable to undergo surgical excision due to patient factors. While in the international statement, there are recommendations for VAE of biopsy-proven benign lesions that patients want removed, there is currently no such clear recommendation from the North America group, and management should be based on institutional standards.

The experts concur that, except for aspiration of simple cysts, a clip marker should be placed in all lesions where biopsy samples are sent for pathology review. Preference for ultrasound visible biopsy markers is particularly recommended for lesions that, due to their location, cannot be visualized with mammography, lesions likely to undergo neoadjuvant chemotherapy (e.g., large size or probable aggressive biology) and may not be visible after treatment, or in very young patients, to minimize radiation involved in imaging clip markers using mammography.

When there is radiological-pathological discordance after CNB or VAB, consensus on further management with either re-biopsy or surgical excision should be reached through multidisciplinary discussion.

In conclusion, an expert radiologist group from the United States and Canada has reviewed the recommendations outlined in “Image-guided biopsy of breast lesions—when to use what biopsy technique” and endorses the statements but notes some regional differences, such as methods for biopsy of axillary lymph nodes and high-risk lesion management. It is also noted that, in contrast to panelist countries, there is no current established practice or recommendations for minimally invasive lesion removal.
